# The Effect of Sampling and Storage on the Fecal Microbiota Composition in Healthy and Diseased Subjects

**DOI:** 10.1371/journal.pone.0126685

**Published:** 2015-05-29

**Authors:** Danyta I. Tedjo, Daisy M. A. E. Jonkers, Paul H. Savelkoul, Ad A. Masclee, Niels van Best, Marieke J. Pierik, John Penders

**Affiliations:** 1 School of Nutrition and Translational Research in Metabolism (NUTRIM), Division Gastroenterology-Hepatology, Maastricht University Medical Center+, Maastricht, The Netherlands; 2 School of Nutrition and Translational Research in Metabolism (NUTRIM), Department of Medical Microbiology, Maastricht University Medical Center+, Maastricht, The Netherlands; 3 School for Public Health and Primary Care (Caphri), Department of Epidemiology, Maastricht University, Maastricht, The Netherlands; University of Camerino, ITALY

## Abstract

Large-scale cohort studies are currently being designed to investigate the human microbiome in health and disease. Adequate sampling strategies are required to limit bias due to shifts in microbial communities during sampling and storage. Therefore, we examined the impact of different sampling and storage conditions on the stability of fecal microbial communities in healthy and diseased subjects. Fecal samples from 10 healthy controls, 10 irritable bowel syndrome and 8 inflammatory bowel disease patients were collected on site, aliquoted immediately after defecation and stored at -80°C, -20°C for 1 week, at +4°C or room temperature for 24 hours. Fecal transport swabs (FecalSwab, Copan) were collected and stored for 48-72 hours at room temperature. We used pyrosequencing of the 16S gene to investigate the stability of microbial communities. Alpha diversity did not differ between all storage methods and -80°C, except for the fecal swabs. UPGMA clustering and principal coordinate analysis showed significant clustering by test subject (p<0.001) but not by storage method. Bray-Curtis dissimilarity and (un)weighted UniFrac showed a significant higher distance between fecal swabs and -80°C versus the other methods and -80°C samples (p<0.009). The relative abundance of *Ruminococcus* and Enterobacteriaceae did not differ between the storage methods versus -80°C, but was higher in fecal swabs (p<0.05). Storage up to 24 hours (at +4°C or room temperature) or freezing at -20°C did not significantly alter the fecal microbial community structure compared to direct freezing of samples from healthy subjects and patients with gastrointestinal disorders.

## Introduction

The gastrointestinal microbiota is the most complex and densely populated ecosystem colonizing the human body. This indigenous microbiota confers several important beneficial functions to its host, including the protection against invading pathogens, stimulation of gut maturation and immune development and homeostasis, and the metabolism of nutrients and xenobiotics. [1]

An altered microbiota composition (dysbiosis) has been associated with a wide range of diseases, such as irritable bowel syndrome (IBS), inflammatory bowel disease (IBD), but also with metabolic diseases such as non-alcoholic fatty liver disease and type-2 diabetes [2–4]. Recent introduction of high-throughput sequencing methods has paved the way for the discovery of key microbial players in the pathophysiology of these diseases. Although differences in composition and metabolic activity have been clearly reported, the causal contribution of such key bacterial species or groups is in most cases not yet clear. This may be due to the relative small study populations, different disease phenotypes, and lack of control for potential confounding factors such as medication, co-morbidity and differences in dietary patterns. Therefore, large well-defined prospective cohorts are required to gain further insight in the role of the microbiota in heterogeneous disease entities. To accurately analyze the fecal microbiota, it is crucial that the sampling and storage procedures do not alter the microbial composition. Therefore, sampling methods that are applicable in large cohorts and do not bias the results are needed for these studies. Although immediate freezing of fecal samples is generally considered the gold standard, this is logistically challenging in large-scale studies. Moreover, thawing of frozen samples during transport is known to have a dramatic effect on DNA integrity [5].

A limited number of studies have examined the effect of different storage methods on the fecal microbiota.

Lauber *et al*. showed that the phylogenetic structure and diversity of bacterial communities in fecal samples were not influenced by storage at room temperature for 14 days compared to aliquots immediately stored at -20°C [6]. However, three other studies demonstrated a significant change in fecal microbiota composition after 24 hour storage at room temperature, but not when frozen within 24 hours [5,7,8].

Apart from storage at room temperature, cooled storage is also feasible in large cohorts. Wu *et al* investigated the storage of fecal samples on ice and showed that such storage for up to 48 hours did not result in significant differences in the fecal bacterial community [9].

The abovementioned studies showed only a small impact of different storage methods on microbiota composition, but the conclusions drawn were based on very small sample sizes including as little as 2 to 4 subjects.[5–9].

Moreover, the vast majority of these studies focused on fecal samples from healthy subjects. It is however conceivable that the effect of sample and storage collection methods differentially affects the microbiota composition in patients, especially in those with altered bowel habits or in patients on medication. Data on the influence of sampling methods on the microbiota of patients with gastrointestinal disorders is lacking.

Along with storage temperature, other user friendly sampling methods should be investigated, which are relatively easy to implement in large cohorts or when including consecutive patients from daily clinical practice. Fecal swabs are routinely used in clinical settings to detect enteropathogens and multidrug resistant Enterobacteriaceae, proving the feasibility of this method [10,11]. Since it is claimed that some of these swabs are also suitable for molecular analysis, it is conceivable that they might also be usable to study the fecal microbiota composition by means of next generation sequencing. However, to the best of our knowledge, no study has explored the fecal swab as a possible sampling method to characterize the fecal microbiota yet.

The aim of the present study is to investigate the effect of different sampling and storage methods on the fecal microbiota composition in both healthy and diseased subjects.

## Material and Methods

### Study population

Ten healthy individuals (HC), 10 IBS outpatients and 8 hospitalized IBD patients (4 ulcerative colitis (UC), 4 crohn’s disease (CD) patients were included in this study. IBS was diagnosed by the Rome III criteria. The IBD diagnosis was based on clinical and endoscopic or radiological findings conform the European Crohn’s and Colitis Organisation (ECCO) guidelines. Disease activity was scored by the validated Harvey Bradshaw index for CD patients and the Simple Clinical Colitis Activity Index for UC patients [12,13].

Exclusion criteria for all subjects were severe co-morbidity and having a stoma or a total colectomy. Healthy subjects were asked to complete the Rome III questionnaire to exclude undiagnosed IBS.

All subjects completed a questionnaire about their age, BMI, smoking status, medical history, medication use.

### Ethical statement

The subjects included in the present study participated in the ongoing IBDSL cohort study (i.e. the IBD patients) or the IBS cohort study (i.e. IBS patients and healthy controls) and gave written informed consent prior to participation. Both study protocols have been approved by the Maastricht University Medical Center+ Committee of Ethics and are executed according to the revised Declaration of Helsinki (59^th^ general assembly of the WMA, Seoul, South Korea, Oct. 2008). The studies have been registered in the US National Library of Medicine (http://www.clinicaltrials.gov, NCT02130349 and NCT00775060, respectively).

### Sample collection

Each subject provided a fresh stool sample in a stool container on site. The consistency of all stool samples was scored using the Bristol stool form scale, ranging from 1 (*i*.*e*. hard lumpy) to 7 (*i*.*e*. watery/liquid stools) [14]. Within 10 minutes upon defecation, the fecal sample was aliquoted and aliquots were immediately stored under different conditions: at -80°C (reference method), at -20°C for 1 week (1wk -20°C), at 4°C for 24 hours (24h +4°C) or at room temperature for 24 hours (24h RT). In addition to the effect of different storage temperatures, a fecal swab (Copan Italia S.P.A., Brescia, Italy), a commercially available transport swab with a preservation medium, often used in clinical practice, (further referred to as FecalSwab) was turned into the stool. The FecalSwabs were stored thereafter at room temperature for 48–72 hours to simulate the scenario of auto-collection by patients and/or study subjects and subsequent transport to the laboratory by mail. Thereafter, all samples were frozen at -80°C until further processing.

### DNA isolation

PSP lysis buffer (Stratec Molecular, Berlin, Germany) was added to a sterile vial containing 0.5 g of 0.1 mm zirconia/silica beards and 4 3.0–3.5 mm glass beads (BioSpec, Bartlesville, USA). Frozen stool aliquots were added to the vials. The samples were homogenized in a MagNA Lyser instrument (Roche, Basel, Switzerland) in three cycles of 1 min. at a speed of 5500 rpm. Samples were kept on ice for one minute in between cycles. DNA isolation was continued using the PSP Spin Stool Kit (Stratec Molecular, Berlin, Germany) according to the manufacturers’ instructions. DNA was finally eluted in 200 μl elution buffer. The DNA quantity and quality was measured by NanoDrop ND-1000 (Thermo Scientific, Wilmington, USA).

Samples with a DNA concentration less than 20 ng or an A_260/280_ less than 1.8 were subjected to ethanol precipitation to concentrate or further purified, respectively, to meet the quality standards. Ten FecalSwab samples with a total DNA content of less than 400 ng were excluded from this research.

### 454 pyrosequencing

A total of 147 samples with a minimal concentration of 20 ng/μl and a 260/280 ratio of at least 1.8 were send to BGI (Hong Kong) for 454 pyrosequencing. Amplicon libraries for pyrosequencing of the 16S rDNA V1–V3 regions were generated using 534F (ATTACCGCGGCTGCTGG) and 27R (AGAGTTTGATCCTGGCTCAG) primers. A key of 10 nucleotides unique for each sample was used. The 454 sequencing run was performed on a GS FLX pyrosequencing system using Titanium chemistry (Roche, Branford, USA).

### Enumeration of *Methanobrevibacter smithii* by qpCR

For those subjects of whom all aliquots were analysed by 454 pyrosequencing, samples were also subjected to an 5’-nuclease based real-time PCR assay for the enumeration of the archeon *M*. *smithii* targeting the 16S rRNA gene as described previously [15]. Amplifications were conducted in a total volume of 25 μL, containing 1× Absolute qPCR Mix (ABgene, Hamburg, Germany), 200 nM of forward and reverse primers, 200 nM TaqMan probe, and 2 μL of tenfold diluted target DNA. The amplification (2 minutes at 50°C, 10 minutes at 95°C, and 42 cycles of 15 seconds at 95°C and 1 minute at 60°C) and detection were conducted with an Applied Biosystems Prism 7900 sequence detection system (Applied Biosystems). Samples with threshold cycle (Ct)-values <40 were considered positive. Log10 DNA copies for a given archaeal species per gram of wet weight faeces was calculated for each stool sample from the Ct-values using a quantification scale ranging from 10–10^7^ target copies.

### Data analysis

The V1–V3 16S rDNA bacterial sequences that were used in this paper have been submitted to the EMBL databases under accession No. PRJEB6765.

To reduce the error rate, raw pyrosequencing reads were passed through quality filters using Mothur version 1.32.1 [16]. Only sequences with a perfect proximal primer fidelity, a minimum average score of 25 over a window size of 50 nucleotides, a read length between 200 and 590, a maximum of one ambiguous base call and a maximum homopolymer length of 6 were retained for further analysis. Subsequent data processing was done by Quantitative Insights Into Microbial Ecology (QIIME) version 1.7 [17].

After de-multiplexing, sequences were clustered by the UCLUST algorithm into operational taxonomic units (OTUs) based on 97% similarity against the Greengenes reference set version May 2013 [18]. Default parameters for UCLUST were applied apart for the following parameters: maxrejects = 100 and stepwords = 16. To reduce the influence of pyrosequencing errors, the creation of de novo OTUs for sequences that did not cluster to reference sequences was disabled.

The following metrics of species richness and diversity within communities (alpha diversity) were determined: observed OTUs (observed richness), Chao1 index (estimated richness), Shannon diversity index and Phylogenetic Diversity (PD) whole tree. Beta diversity or diversity shared across samples was determined by the unweighted and weighted UniFrac distance and Bray-Curtis dissimilarity (BC) at a rarefaction depth of 3445 seq/sample. [19].

### Statistical analysis

In order to examine the potential loss in species richness/diversity during storage, pairwise comparisons were made for the alpha diversity metrics between the aliquots stored according to the reference method (direct storage at -80°C) and each of the other storage methods by means of the Wilcoxon signed rank test.

To determine the effect of storage on the shared diversity between samples, beta diversity metrics (Bray-Curtis dissimilarity, unweighted UniFrac and weighted UniFrac) were calculated for the aliquots stored at -80°C versus each of the other storage methods (*i*.*e*. -80°C-1wk 20°C, -80°C-24h +4°C, -80°C-24h RT, and -80°C-FS). Subsequently these distances (within subjects across sampling methods) were tested by means of the Wilcoxon signed rank test. The Mann-Whitney U test was used to compare the distances of the reference methods between subjects.

Statistical differences in community structure among test subjects and sampling and storage method were tested using PERMANOVA. The G-test was used to test associations between OTUs presence and storage method. To correct for multiple testing a false discovery rate (FDR) of 0.25 was used as a cut off value.

To examine whether storage methods had a significant impact on the recovery of oxygen sensitive microorganisms, pairwise comparisons of the relative abundance of *Ruminococcus*, *Faecalibacterium*, *Roseburia* and *M*. *Smithii* was performed by the Wilcoxon signed rank test.

In accordance, differences in the relative abundance of *Enterobacteriaceae* was also examined by the Wilcoxon signed rank test since the Cary-Blair medium of the FecalSwab is known to enhance the recovery of enteropathogenic bacteria [20].

Statistical analysis were performed using SPSS version 19 and QIIME 1.7.

## Results

### Study population

A total of 28 study subjects, 10 healthy individuals, 10 IBS patients and 8 IBD patients (18–71 years) were included in this study. Half of the IBS patients were diagnosed with diarrhea pre-dominant IBS (n = 5), followed by mixed IBS (n = 4) and constipation pre-dominant IBS (n = 1). None of the study subjects used probiotics, whereas one IBS patient followed a strict vegetarian as well as a gluten free diet at the time of the study. Among the IBD patients, 4 patients suffered from Crohn’s disease and 4 patients had ulcerative colitis. All except one IBD patient were experiencing an exacerbation at the time of fecal sampling. Baseline characteristics of all study participants are presented in Table 1.

**Table 1:** Characteristics of study population.

	HC (n = 10)	IBS (n = 10)	IBD (n = 8)
Age (years)	25.5 (23–44)	51.5 (23–71)	55.5 (20–67)
Male (%)	60	60	37.5
BMI	21.3 (19.2–24.8)	23.8 (18.1–34.7)	27.6 (35.3–22.7)
Smoker (%):			
- Currently	0	20	0
- Stopped	10	10	50
- Never	90	70	50
Stool consistency^6^ (Bristol Stool):			
- Type 1–2	0	3	0
- Type 3–4	10	3	0
- Type 5–7	0	4	8
Disease phenotype^1^	n.a.	IBS-D: 5	4 CD
		IBS-C: 1	4 UC
		IBS-M: 4	
Disease location	n.a.	n.a.	colonic: 1 left sided:3 ileocolonic: 2 pancolitis:1
Medication (n)^2^	3	8	9
	Birth control: 2	Birth control: 2	TNF-α: 3
	Ironsupplement and asthma medication:1	Motility: 3	Thiopurine: 2
		Antidepressant: 4	Aminosalicylate: 2
		PPI: 1	Methotrexate
		Analgesics: 3	Glucocorticoid: 6
			Antibiotics: 2
Abdominal surgery	0	4^3^	2^4^
Active disease^5^	n.a.	n.a.	CD: 4/4 UC: 3/4

Continuous variables are presented as median and range.

^1^: CD: Crohn’s disease, UC: ulcerative colitis, IBS-D: diarrhea predominant IBS, IBS-C: constipation predominant IBS, IBS-M: mixed IBS

^2^: Patients took multiple medications.

^3^: 2 subjects had a hysterectomy, 1 subject had a sigmoid resection, 1 subject underwent an appendectomy

^4^: 1 subject had a hysterectomy, 1 subject had a hysterectomy, appendectomy and illeocecal resection

^5^: Active disease if Harvey-Bradshaw Index (HBI)>4 (CD) or Simple Clinical Colitis Activity Index (SCCAI)>3 (UC)

^6^: Type 1–2: Type 1–2: hard/lumpy stool. Type 3–4: sausage shaped stool (with cracks or being smooth/soft). Type 5–7: soft blobs to watery/liquid consistency

### 454 sequencing

A complete set of samples (*i*.*e*. immediately stored at -80°C, stored at -20 for 1 week, stored at +4°C for 24 hours, stored at room temperature and a FecalSwab) was obtained from 28 individuals. Ten out of 28 FecalSwab samples were excluded for 16S V1–V3 library construction due to an insufficient DNA yield.

**Fig 1:**
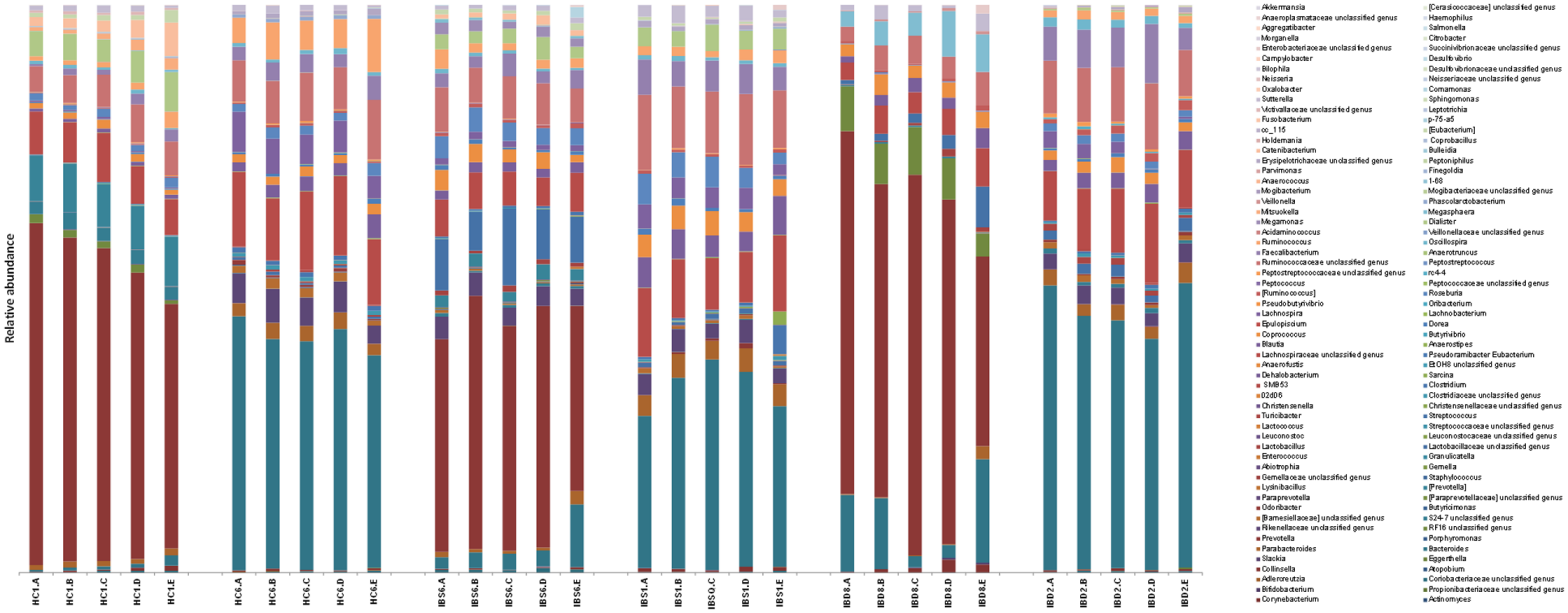
Relative distribution of bacterial taxa at genus level per storage or sampling method for a representative set of test subjects (2 healthy controls, 2 IBS and 2 IBD patients). Letters indicate the storage method (A = -80°C, B = 1wk -20°C, C = 24h +4°C, D = 24h RT, E = FecalSwab).

In total, 1,825,747 raw sequences were obtained from sequencing all remaining samples. After quality filtering and binning, a total of 927,825 sequences with an average of 7,137 sequences per sample (range 1,066–25,887 sequences/sample) remained for further analysis. A total of 6,105 OTUs were detected with a mean Good’s coverage of 92 ± 0.03%. The microbiota of the fecal samples stored according to the reference method was dominated by Bacteroidetes (mean 50.4%, SD 0.17%) and Firmicutes (mean 42.1%, SD 0.17%), followed by Proteobacteria (mean 4.6%, SD 5.3%). No significant differences in the relative abundance of the dominant phyla were found between the reference method and other sampling and storage methods. The microbial profile at the genus level within subjects was rather similar for aliquots stored under the different conditions (Fig 1).

### Effect of storage methods on diversity and richness

**Fig 2:**
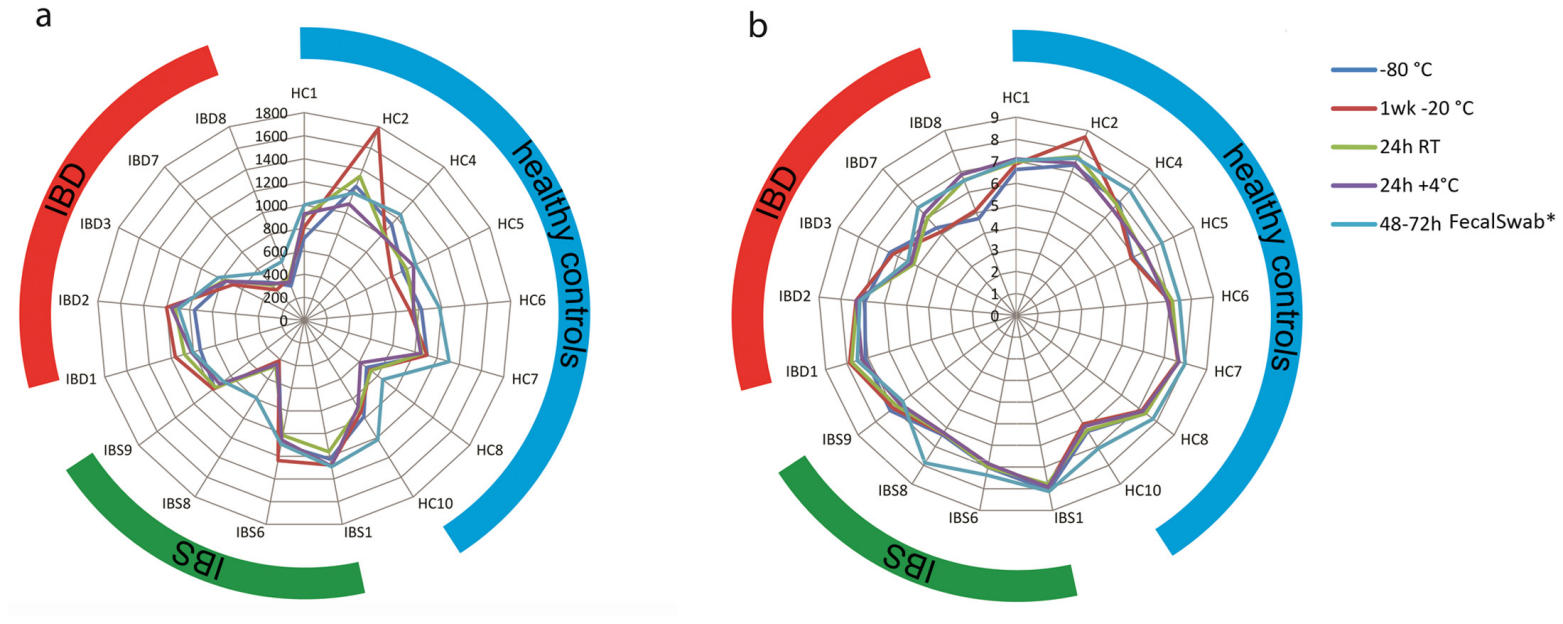
Effect of storage methods on the alpha diversity. Chao 1 richness estimate (a) and Shannon index (b) is shown at the y axis and test subjects are shown at the x axis. The blue, green and red bands indicate healthy test subjects, IBS and IBD patients respectively. Only test subjects (17/28) with a complete set of samples (5 different sampling and storage methods) available for analysis are shown. *p<0.01 compared with -80°C as determined by the wilcoxon signed rank test.

The Chao1 richness estimates and Shannon diversity indices did not differ statistically significantly between samples stored directly at -80°C and the aliquots from the same samples stored at -20°C for one week (p = 0.829 and p = 0.456, respectively), 4°C for 24 hours (p = 0.139 and p = 0.838, respectively) and room temperature for 24 hours (p = 0.946 and p = 0.466 respectively). The FecalSwab however, showed a statistically significantly higher Chao1 estimate and Shannon index (p<0.01 and p<0.01, respectively) than the reference method. The Chao1 richness and Shannon diversity indices for subjects of whom all aliquots were available for analysis (n = 17) are depicted in Fig 2. Of note, the -20°C sample of HC2 showed higher Chao1 and Shannon estimates as compared to the other aliquots of this subject. No such differences were observed for the other subjects.

Pairwise comparisons of other alpha diversity indices (observed richness and PD-whole tree) also showed a significantly higher diversities in the FecalSwab as compared to the aliquots stored at -80°C, but no significant differences were found between this reference method and other storage methods (Table 2).

**Table 2:** Effect of sampling and storage methods on alpha diversity metrics.

Diversity indices	Sampling & storage methods				
	-80°C (n = 28)	1w -20°C (n = 27)	24h RT (n = 28)	24h +4°C (n = 28)	48–72h FecalSwab (n = 18)
**Observed species**	540.1 (112.5–811.2)	545.5 (93.8–995.9)	549.2 (95.3–830.6)	529.1 (90.6–791.4)	593.9* (280.8–746.9)
**Chao1**	955.8 (206.0–1457.3)	935.2 (143.6–1780.6)	963.8 (180.1–1525.6)	936.36 (130.1–1440.3)	1042,62* (544.4–1308.4)
**Shannon**	7.0 (4.4–8.4)	6.9 (4.1–8.7)	7.0 (4.1–8.4)	6.9 (4.4–8.5)	7.4* (5.5–8.1)
**PD whole tree**	27.5 (8.6–37.3)	27.5 (6.8–46.0)	28.4 (7.8–37.7)	27.6 (7.0–37.2)	30.0* (14.5–37.3)

Median and range are shown in the table. (*p<0.05 compared to -80°C).

### Effect of storage on the fecal microbial structure

**Fig 3:**
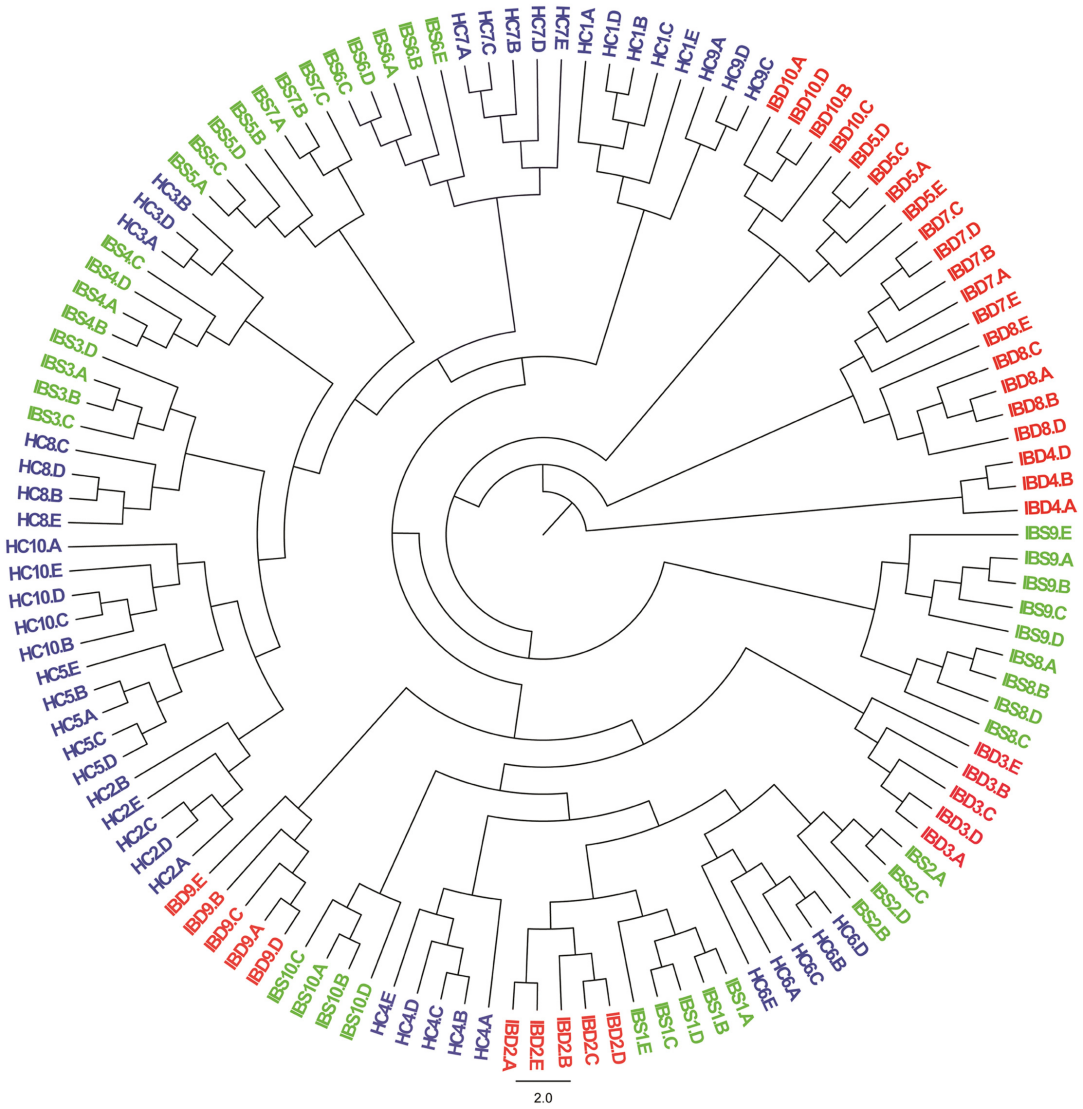
UPGMA tree based on unweighted UniFrac distance. All subjects (n = 28) are included. Letters indicate the storage method (A = -80°C, B = 1wk -20°C, C = 24h +4°C, D = 24h RT, E = FecalSwab). Samples of healthy subjects (HC), IBS and IBD patients are written in blue, green and red, respectively.

UPGMA clustering based on unweighted pair-wise UniFrac distances between all samples resulted in separation of the samples based on test subjects as shown in Fig 3. In line with the above findings, principal coordinate analysis of unweighted UniFrac distance showed a strong clustering of the samples based on test subjects, although the FecalSwabs seems to deviate most from its original subject specific cluster (S1 Fig, S2 Fig and S3 Fig). Principal coordinate analysis of the weighted UniFrac distances and Bray-Curtis dissimilarity showed similar results (S4–S9 Figs). Moreover, applying PERMANOVA revealed that samples between subjects were statistically significantly different from each other (p<0.001 for unweighted UniFrac, weighted UniFrac and Bray-Curtis). No significant clustering was found based on the different sampling and storage methods by PERMANOVA.

**Fig 4:**
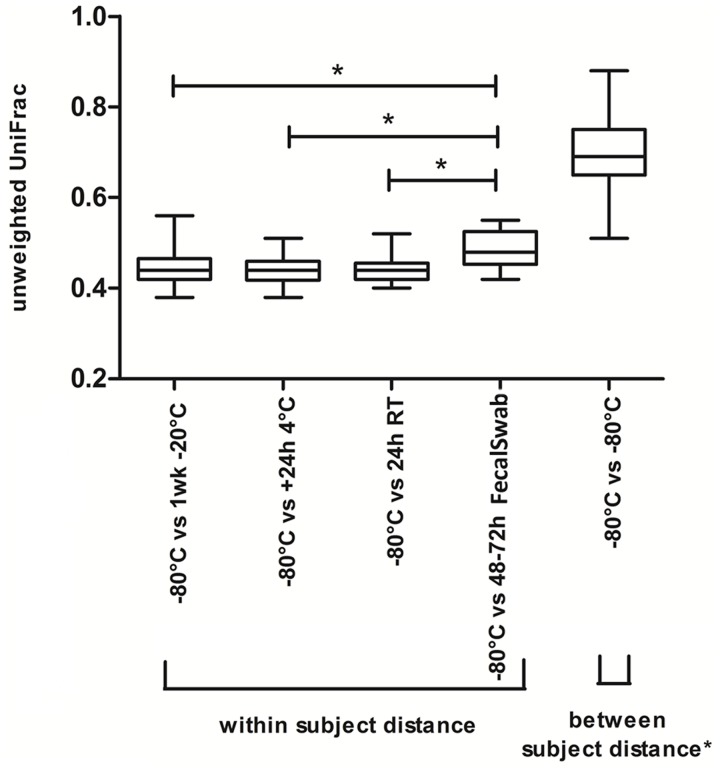
Box-and-whisker plot of unweighted UniFrac distance between reference method and other sampling and storage methods. Whiskers express the maximum and minimum values. *indicate a significant difference (p<0.01).

To examine the impact of individual storage methods in more detail, we subsequently examined the beta diversity distances between the reference storage method and each of the other storage methods. The median and interquartile unweighted UniFrac distances are depicted in Fig 4.

The between-subject unweighted UniFrac distance of all samples stored at -80°C (reference method) was statistically significantly larger than the within-subject unweighted UniFrac distances between the reference method and each of the other storage methods (p<0.05). However, paired comparisons demonstrated a statistically significantly larger unweighted UniFrac distance between the reference method and the FecalSwab as compared to the distance between the reference method and all other storage methods as seen in Fig 4. Pairwise comparisons of the weighted UniFrac distance and Bray-Curtis dissimilarity also showed that the beta diversity distances between the FecalSwab and the reference method is statistically significantly larger compared to the reference method and all other storage methods (S10 and S11 Figs). The median unweighted UniFrac distances of -20°C, +4°C and RT versus the reference method were comparable between each other (Fig 4), implying that aliquots stored under these conditions were equally similar to samples stored directly at -80C. This is also shown when pairwise comparisons were made of the weighted UniFrac and Bray-Curtis dissimilarity (S10 and S11 Figs). The effect of the different storage methods was not significant in the subgroups of healthy subjects, IBS and IBD patients.

### Effect of sampling and storage on the presence and relative abundance of specific bacterial taxa

No association between the presence of specific bacterial taxa and the different storage methods was found as examined by applying the G- test with a cut-off value of q = 0.25.

**Fig 5:**
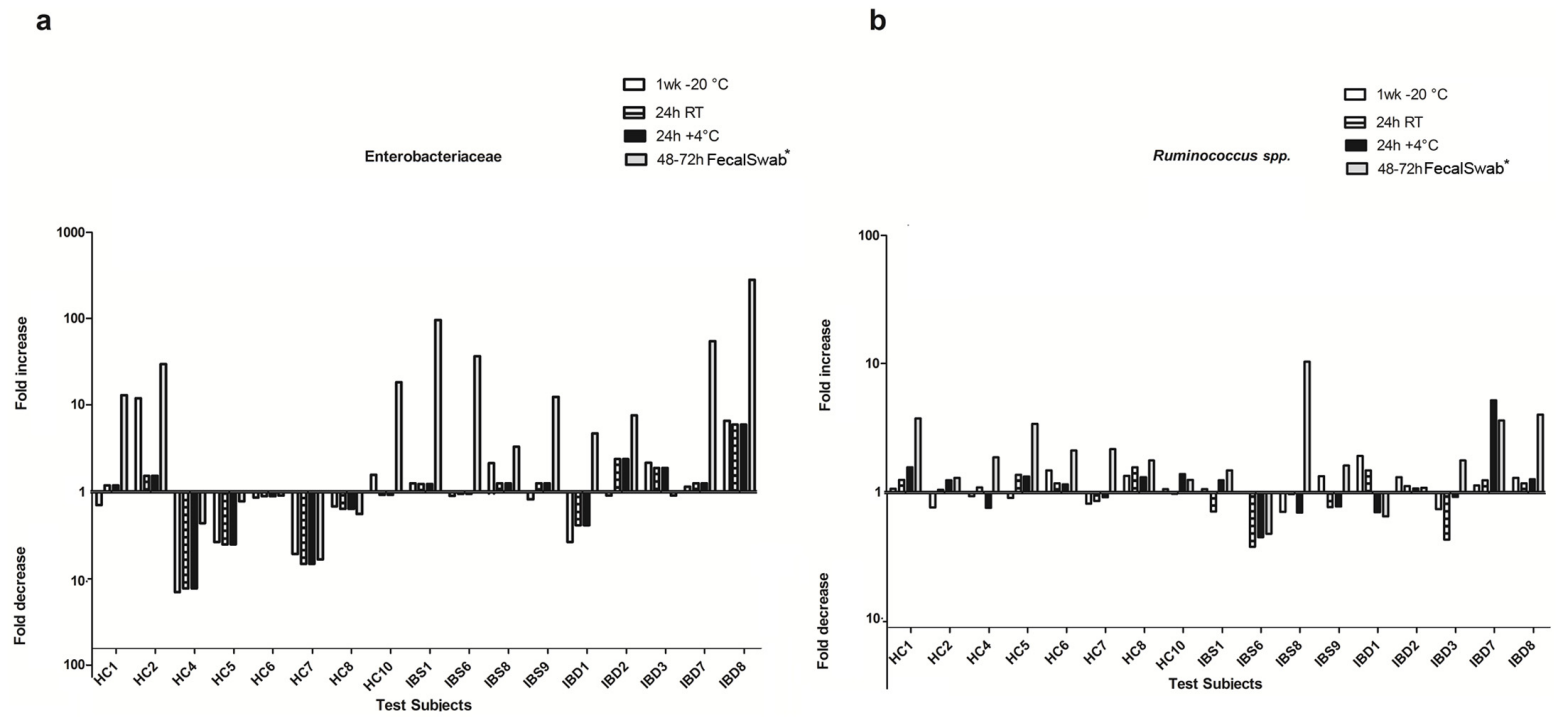
Fold change of relative abundance of Enterobacteriaceae (a) and *Ruminococcus spp*. (b) compared to -80°C. Only test subjects (17/28) with a complete set of samples (5 different sampling and storage methods) available are shown. Fold changes are shown at the y axis (logarithmic scale). * Relative abundance in FecalSwabs was significantly higher as compared to reference storage method (p<0.05 as determined by Wilcoxon signed rank test).

Subsequently, the relative abundances of *Faecalibacterium*, *Roseburia* and *Ruminococcus* were examined since these species are known to be extremely sensitive to oxygen and thus different sampling and storage methods. No significant differences were found between samples stored at -80°C and the other sampling and storage methods in the relative abundance of *Faecalibacterium* and *Roseburia*. Remarkably, the relative abundance of *Ruminococcus* showed a significant increase in the FecalSwab compared to the -80°C samples (p<0.05), but not in the other storage methods (Fig 5). In addition to the extreme oxygen-sensitive species, the relative abundance of Enterobacteriaceae was examined. Paired comparisons showed a significantly higher abundance of Enterobacteriaceae in FecalSwab (p<0.05), but not in the other storage methods, when compared to the reference method.

Quantification of the archeon *M*. *smithii* revealed that in case of a positive result for the aliquot stored at -80°C (n = 9/18), all other aliquots (20°C, +4°C and RT) gave a positive PCR as well. Morover, the concentration of *M*. *smithii* in positive reference samples (median = 8.54; range 6.70–10.63 log10 DNA copies/g faeces) did not differ from the concentration in the other aliquots. However, the FecalSwab samples were PCR negative in the majority of subjects of whom the reference sample was positive (n = 7/9) due to the strong diluting effect of the preservation buffer.

## Discussion

The aim of this study was to investigate the effect of different sampling and storage methods on the stability of the fecal microbiota composition in healthy and diseased subjects. Previous studies have investigated the effect of various storage temperatures on the fecal microbiota composition and showed overall no significant differences in richness, diversity and clustering. These studies were however based upon a limited number of subjects, included only healthy individuals or did not report the health status of included subjects. As such, the generalizability of their results and conclusions is unclear [5–9]. Our study included both healthy as well as diseased subjects; diagnosed with common GI disorders that are reported to be associated with the intestinal microbiota, have differences in fecal consistency and use a variety of medications which enhances the heterogeneity of our study population and thereby the external validity of our results. No significant differences were found in the amount of observed OTUs, richness and diversity between the reference storage method and other methods. Similar results were observed in the total study population, as well as in the subgroups of healthy subjects, IBS and IBD patients and did not differ with regard to stools consistency (i.e. ranging from constipation to diarrhea type stools).

Our results are in line with most previous studies, which showed no significant change in the overall microbiota composition between frozen fecal samples and samples stored at room temperature or 4°C for 24 hours [5–9]. Only one previous study compared the microbial richness of directly frozen samples and samples stored at room temperature up to 24 hours and showed no statistically significant differences [8], which is in accordance with our findings. Previous studies were mainly performed in small numbers of healthy subjects or with unknown health status. We showed that the findings are also applicable to patients with different disease states, medication use and/or fecal consistencies. In contrast to the study of Roesch et al, which included four healthy subjects, we did not observed a decrease in the relative abundance of the extreme oxygen sensitive species *Faecalibacterium* and *Ruminococcus* or an increase in the relative abundance of Enterobacteriaceae in the fecal samples stored at room temperature for 24 hours as compared to the reference method in our cohort of 28 study objects. In addition to these results, we found that the relative abundance of extreme oxygen sensitive species *Roseburia* and the Firmicutes/Bacteroides ratio was not significantly different between the storage methods and reference method. Our results indicate only a minimal effect of the investigated storage methods on the fecal microbiota composition from the total population and all subgroups investigated. This observation is further supported by the UPGMA clustering and principal coordinate analyses, which showed that the fecal aliquots clustered based on test subject and not storage methods. Although the distances in beta diversity are relatively small and comparable between each and every storage method, we still recommend using a single standardized method within one study to minimize any potential sampling bias.

The variations seen within individuals are at least partly explained by the sequencing depth. We are aware that our sequencing depth is not ultradeep, therefore low abundant OTUs will not always be detected. However, the conclusions of our study did not change when we performed a subanalysis with the samples that had a higher sequencing depth (rarefaction at 5,000 seq/sample) (S12–S14 Figs and S1 Table).

In the present study FecalSwab samples were included to examine whether this sampling strategy, often used in the clinical setting, is also suitable for microbiota analysis. The FecalSwabs were stored for 48–72 hours at room temperature in modified Cary-Blair medium, which promotes survival of obligate anaerobes by the low oxidation reducing potential and is used for the isolation of enteric pathogens [20]. FecalSwabs showed a significantly higher microbial richness and diversity. This might be explained by a slight growth advantage of certain low abundant bacterial taxa, particularly within the significantly enriched Enterobacteriaceae and Ruminococcaceae families, in this medium. Such low abundant taxa, undetected at the applied sequencing depth in the other aliquots, could then rise above the detection limit. Although FecalSwabs still strongly clustered to the other aliquots of the same subject, this could also explain why the microbial community structure of the FecalSwabs showed a larger distance (Bray-Curtis dissimilarity and UniFrac distances) to the reference method than the other storage methods. The use of fecal swabs seems to be the most user friendly method to obtain fecal samples, however, in this study 10 out of 28 FecalSwab samples yielded insufficient DNA quantity and could therefore not be sequenced. When using FecalSwabs, an adapted DNA isolation protocol (*e*.*g*. not depending on column-based purification) is recommended to retain highest possible DNA yield. The results of the FecalSwab are only applicable to this particular swab and other transport swabs containing Cary-Blair medium and should not be extrapolated to swabs using different transport medium. It is important to note that within or between studies, the results of the FecalSwab should not be compared with other feces collection and storage methods due to the variations in the microbiological structure between these sampling methods. To our best knowledge this is the first study that investigates the effect of different sampling and storage methods by means of pyrosequencing within a rather large, heterogeneous group. By including healthy subjects and those with a GI disorder, the results drawn from this study can be generalized to other populations. The results and conclusions are only applicable for studies on the taxonomic composition of the fecal microbiota. Metagenomics, metatranscriptomics, metaproteomics, and metametabolomics are becoming increasingly important in the study and understanding of the human GI microbiota. Metabolites, proteins and especially mRNA are known to be susceptible to enzymatic degradation. Therefore, studies on the impact of different sampling and storage methods on the stability of the metagenome, metametabolome, metatranscriptome and metaproteome are also warranted.

Based on our and previous observations, we conclude that fecal samples stored at -20°C for 1 week, at +4°C for 24 hours and at room temperature for 24 hours retained highly similar bacterial structure at DNA level as fecal samples stored in the -80°C within 10 minutes. Direct freezing of the feces (e.g. in the home freezer of participating subjects) increases the potential for additional analyses (eg. metatranscriptomics, metaproteomics), but thawing of the samples during transport should be prevented at any cost [8]. If direct freezing is not feasible within the setting of a study, we recommend storing feces at either room temperature or at +4°C (in a refrigerator) for a maximum of 24 hours.

When using the FecalSwab as a sampling method, the isolation procedure should be modified to ensure high DNA quality and quantity. Moreover, transport time (storage at room temperature) should be limited as the microbial community structures in the FecalSwabs (after 48–72h at room temperature) deviated most from the original microbial community structure. Homogenization of the feces prior processing appears not necessary since recent work showed that homogenization does not alter the fecal microbiota [21]. We recommend applying a single sampling and storage method within a study to prevent potential bias in the results.

## Supporting Information

S1 Fig Principal coordinate analysis based on unweighted UniFrac distance for healthy subjects colored based on subject (a) and storage method (b). (TIF)

S2 Fig Principal coordinate analysis based on unweighted UniFrac distance for IBS patients colored based on subject (a) and storage method (b). (TIF)

S3 Fig Principal coordinate analysis based on unweighted UniFrac distance for IBD patients colored based on subject (a) and storage method (b). (TIF)

S4 Fig Principal coordinate analysis based on Bray-Curtis distance for healthy subjects colored based on subject (a) and storage method (b). (TIF)

S5 Fig Principal coordinate analysis based on Bray-Curtis distance for IBS patients colored based on subject (a) and storage method (b). (TIF)

S6 Fig Principal coordinate analysis based on Bray-Curtis distance for IBD patients colored based on subject (a) and storage method (b). (TIF)

S7 Fig Principal coordinate analysis based on weighted UniFrac distance for healthy subjects colored based on subject (a) and storage method (b). (TIF)

S8 Fig Principal coordinate analysis based on weighted UniFrac distance for IBS patients colored based on subject (a) and storage method (b). (TIF)

S9 Fig Principal coordinate analysis based on weighted UniFrac distance for IBD patients colored based on subject (a) and storage method (b). (TIF)

S10 Fig Box and whisker plot of weighted UniFrac distance between reference method and other sampling and storage methods. (*p<0.05).
(TIF)

S11 Fig Box and whisker plot of Bray-Curtis dissimilarity between reference method and other sampling and storage methods. (*p<0.05).
(TIF)

S12 Fig Box and whisker plot of Bray-Curtis dissimilarity between reference method and other sampling and storage methods at a sampling depth of 5,000 seq/sample. (*p<0.05).
(TIF)

S13 Fig Box and whisker plot of unweighted UniFrac distance between reference method and other sampling and storage methods at a sampling depth of 5,000 seq/sample. (*p<0.05).
(TIF)

S14 Fig Box and whisker plot of weighted UniFrac distance between reference method and other sampling and storage methods at a sampling depth of 5,000 seq/sample. (*p<0.05).
(TIF)

S15 Fig Effect of storage methods and stool consistency on the alpha diversity. Chao 1 richness estimate (a) and Shannon index (b) is shown at the y axis and test subjects are shown at the x axis. The blue and red bands indicate subjects with normal stool and subjects with diarrhea respectively. Only test subjects (17/28) with a complete set of samples (5 different sampling and storage methods) available for analysis are shown. *p<0.05 comparison diarrhea versus normal stool. ^1^Bristol stool scale 3–4. ^2^Bristol stool scale 5–7.
(TIF)

S1 Table Effect of sampling and storage methods on alpha diversity metrics at a sampling depth of 5000 sequences/sample. Median and range are shown in the table. (*p<0.05 compared to -80°C).
(DOCX)
