# Supplementary figures and images for: The Effect of Sampling and Storage on the Fecal Microbiota Composition in Healthy and Diseased Subjects
Source: PLoS One. 2015 May 29;10(5):e0126685. doi: 10.1371/journal.pone.0126685 (PMC4449036; doi:10.1371/journal.pone.0126685)

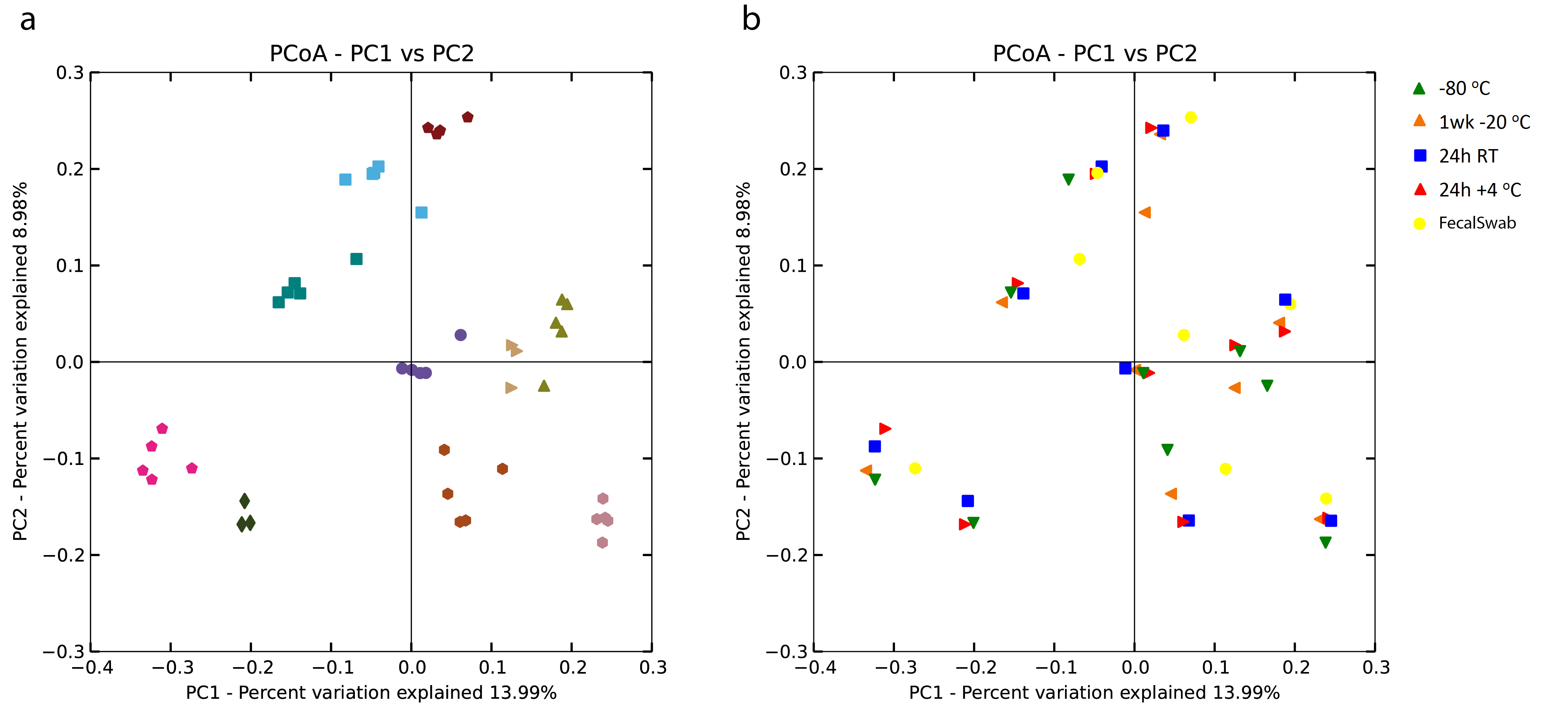

Supplement: S1 Fig — (TIF) [file pone.0126685.s001.tif]

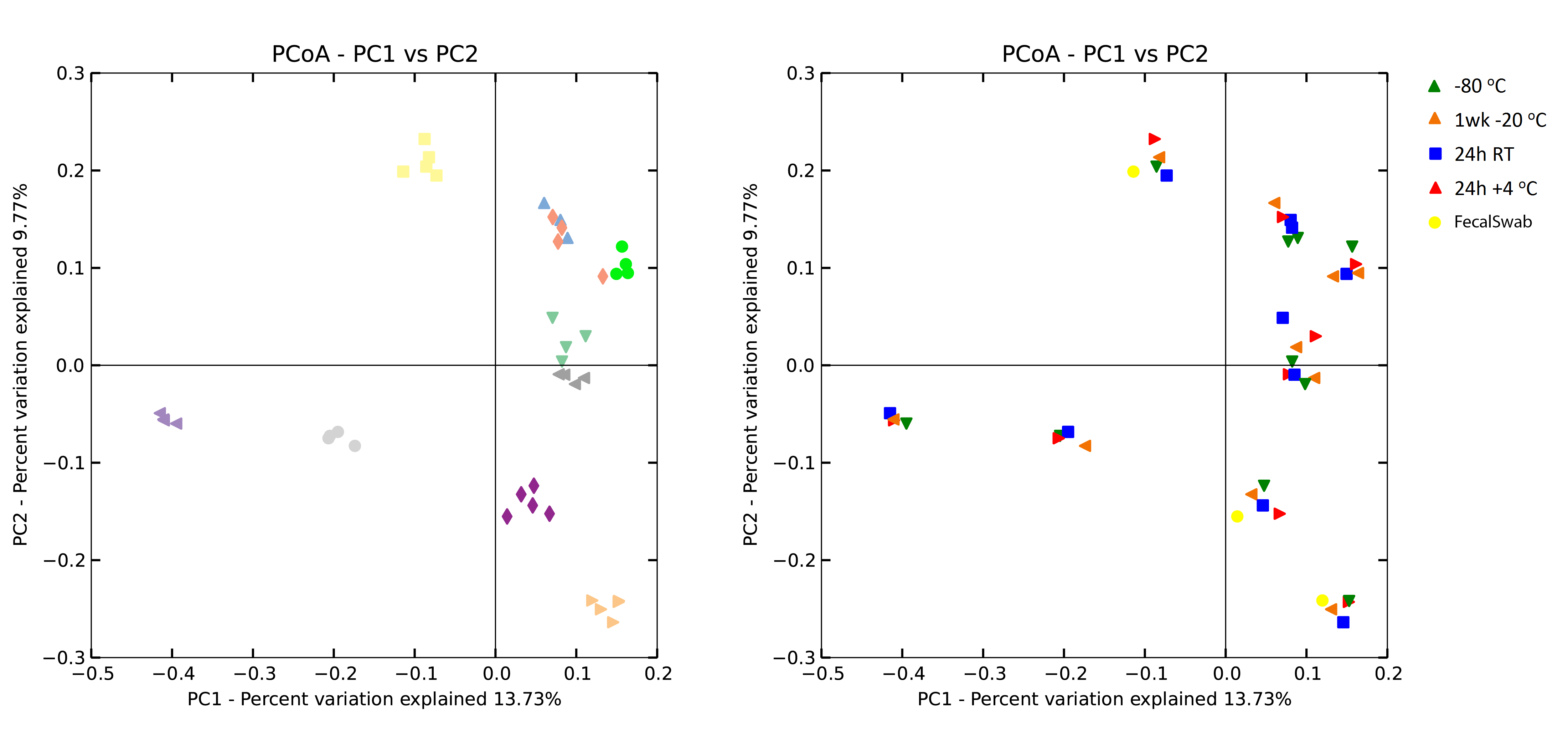

Supplement: S2 Fig — (TIF) [file pone.0126685.s002.tif]

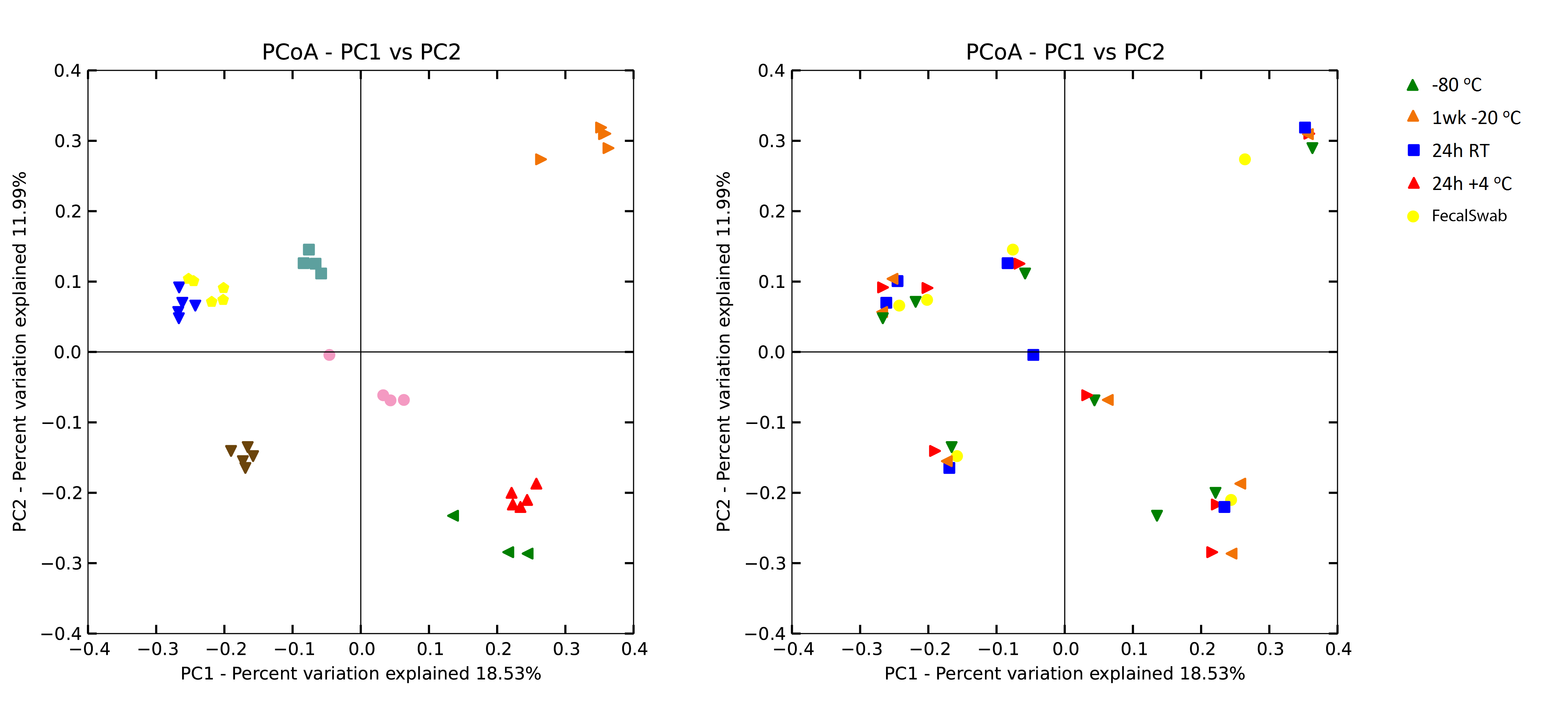

Supplement: S3 Fig — (TIF) [file pone.0126685.s003.tif]

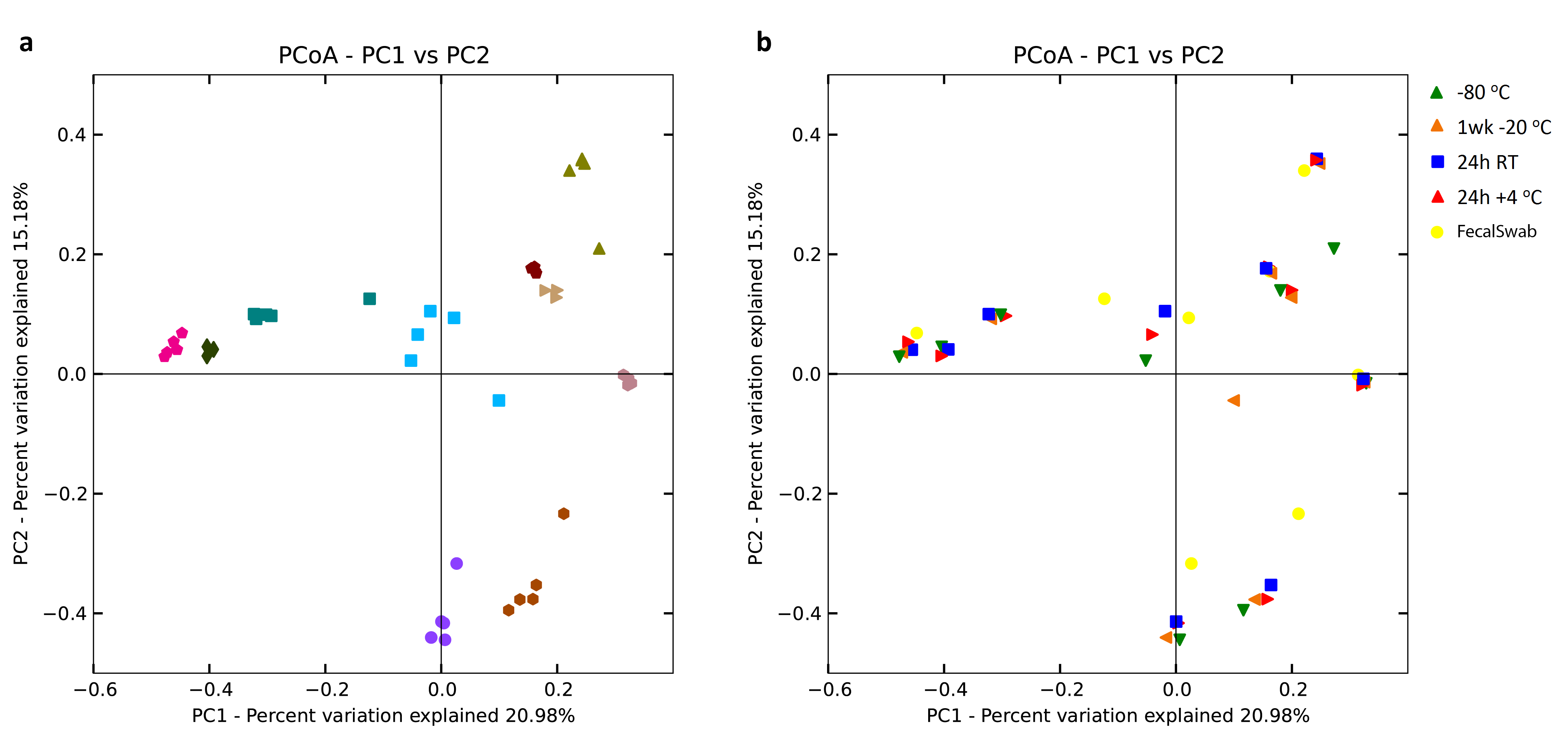

Supplement: S4 Fig — (TIF) [file pone.0126685.s004.tif]

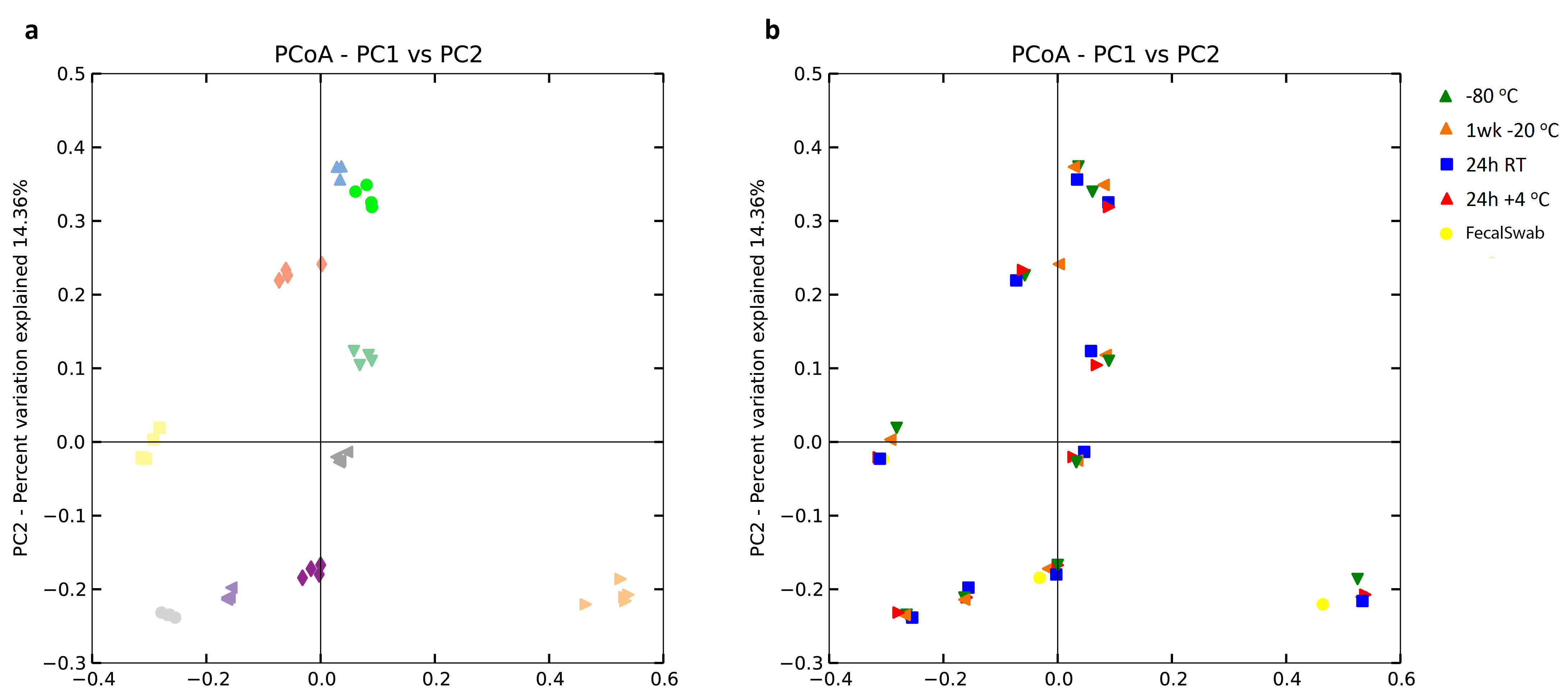

Supplement: S5 Fig — (TIF) [file pone.0126685.s005.tif]

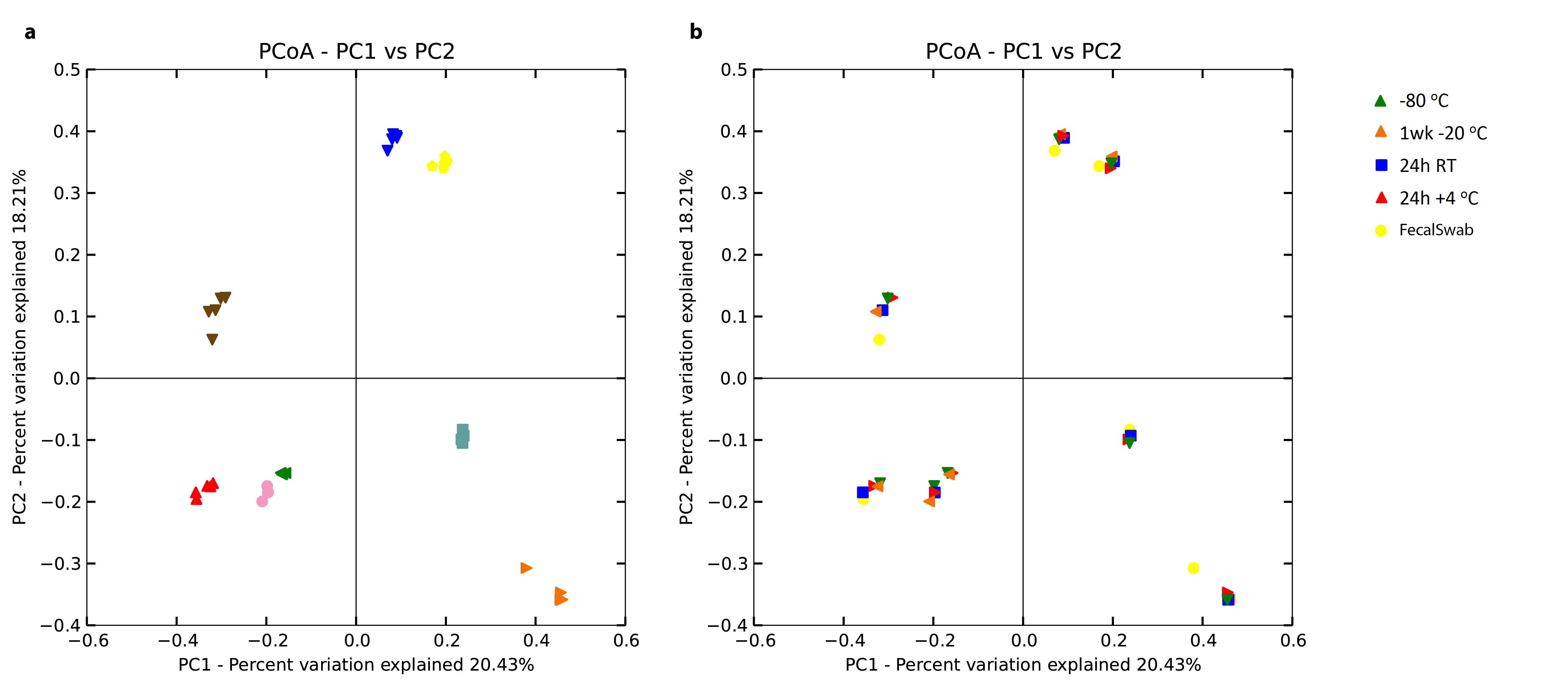

Supplement: S6 Fig — (TIF) [file pone.0126685.s006.tif]

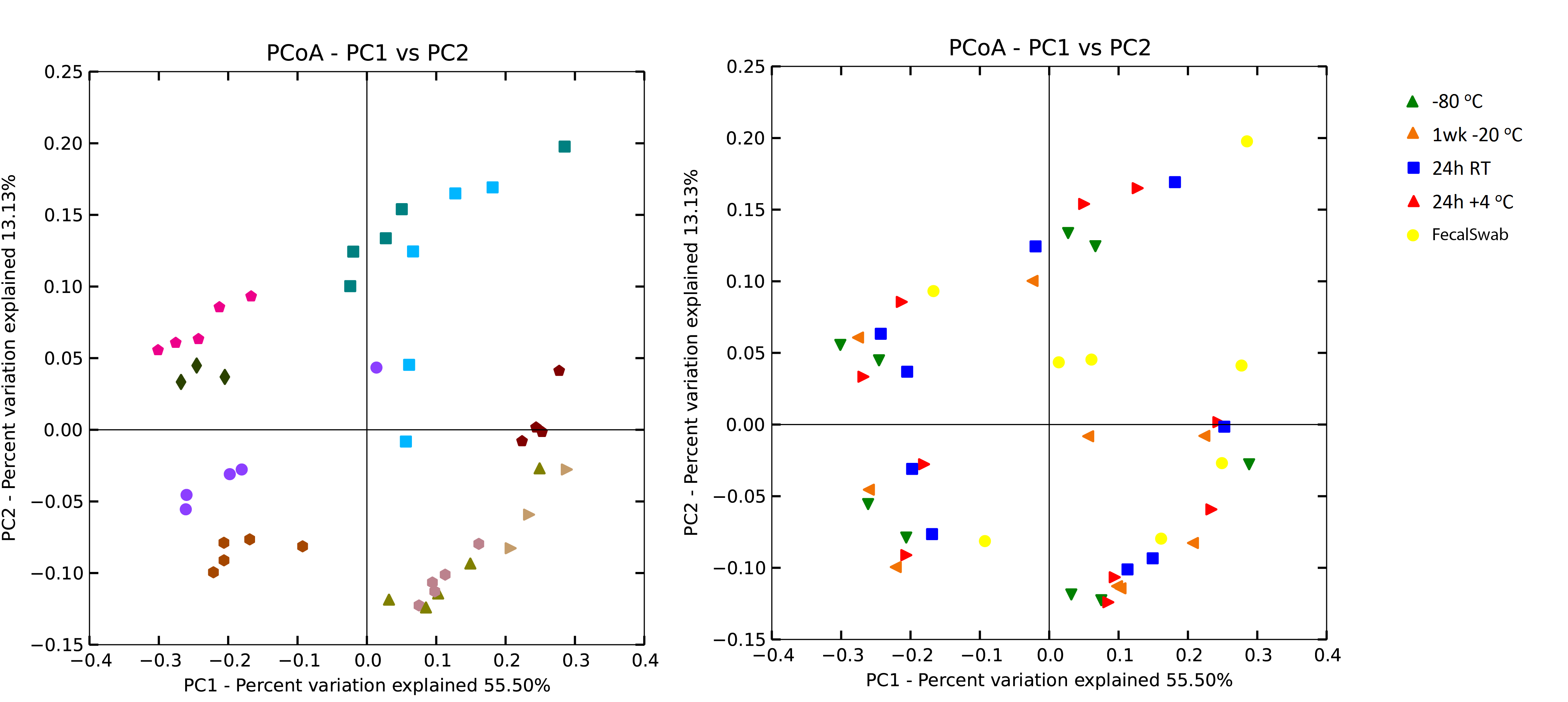

Supplement: S7 Fig — (TIF) [file pone.0126685.s007.tif]

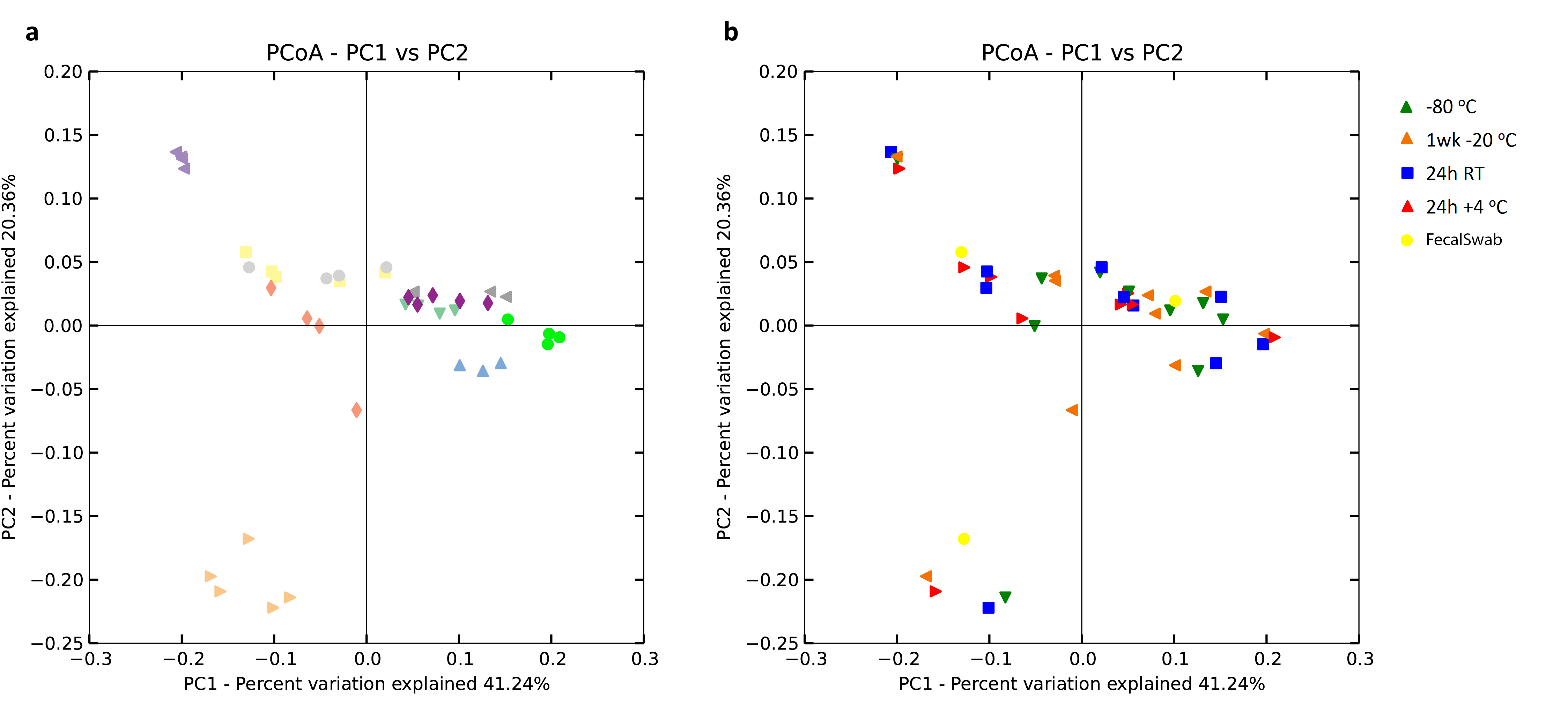

Supplement: S8 Fig — (TIF) [file pone.0126685.s008.tif]

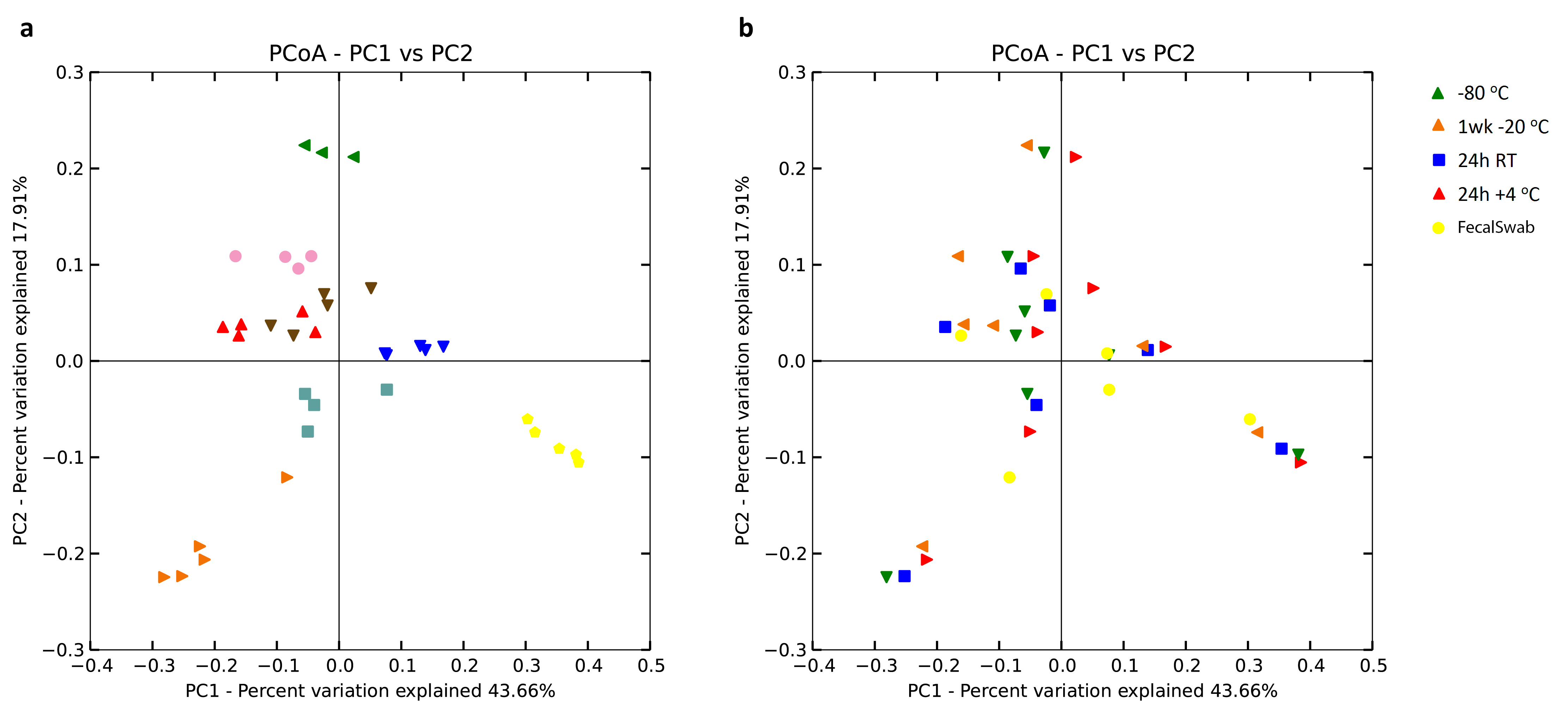

Supplement: S9 Fig — (TIF) [file pone.0126685.s009.tif]

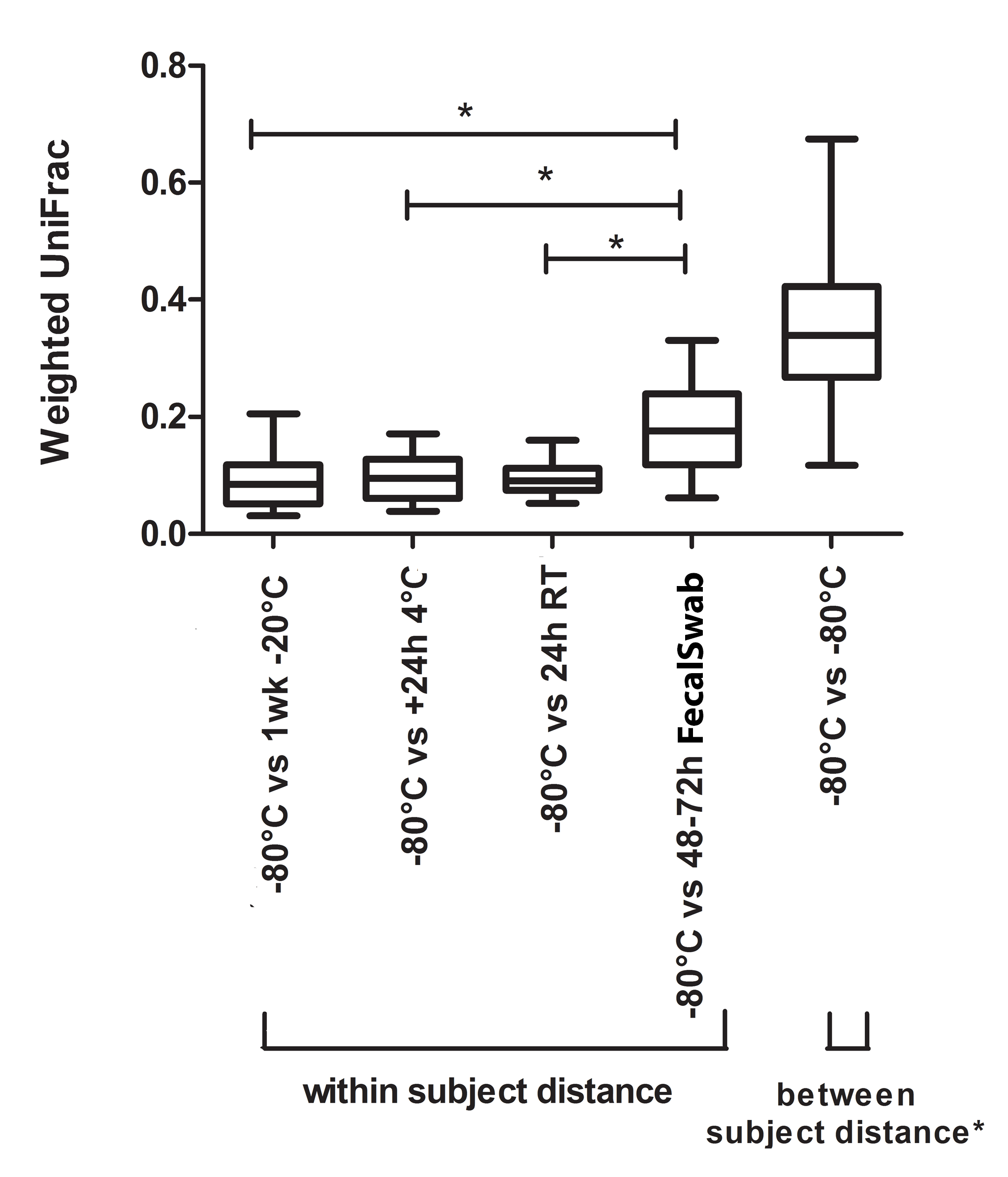

Supplement: S10 Fig — (*p<0.05). (TIF) [file pone.0126685.s010.tif]

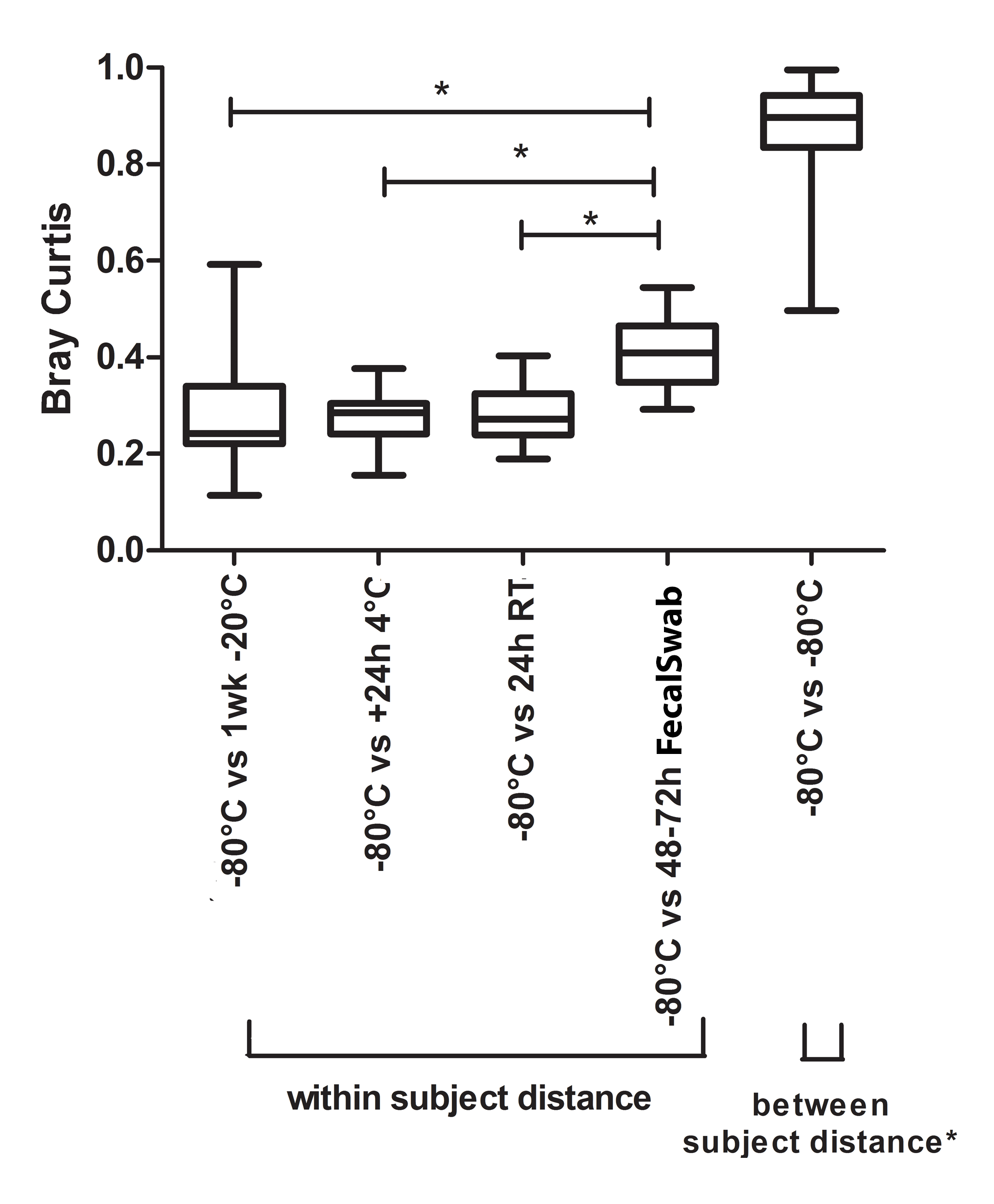

Supplement: S11 Fig — (*p<0.05). (TIF) [file pone.0126685.s011.tif]

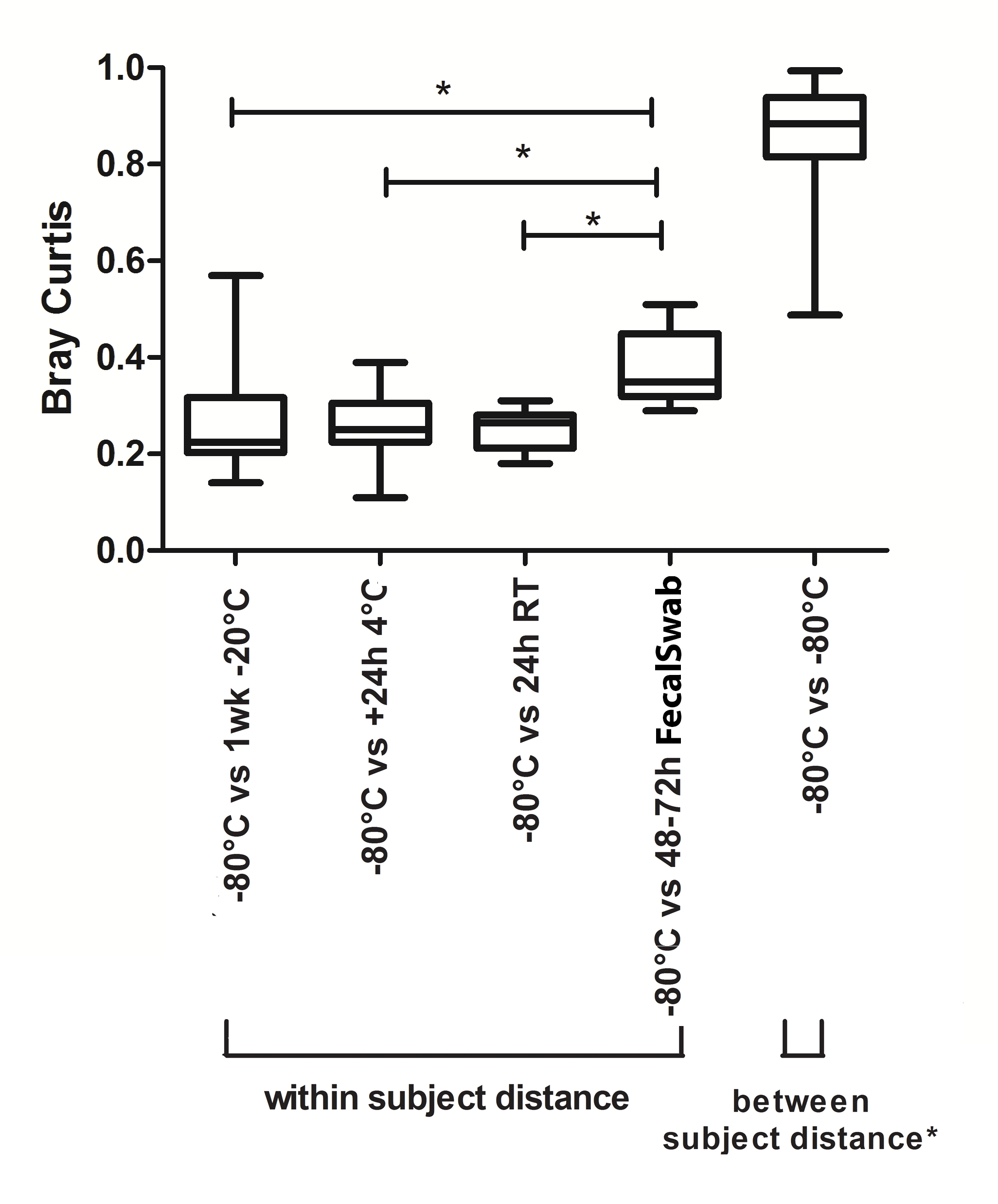

Supplement: S12 Fig — (*p<0.05). (TIF) [file pone.0126685.s012.tif]

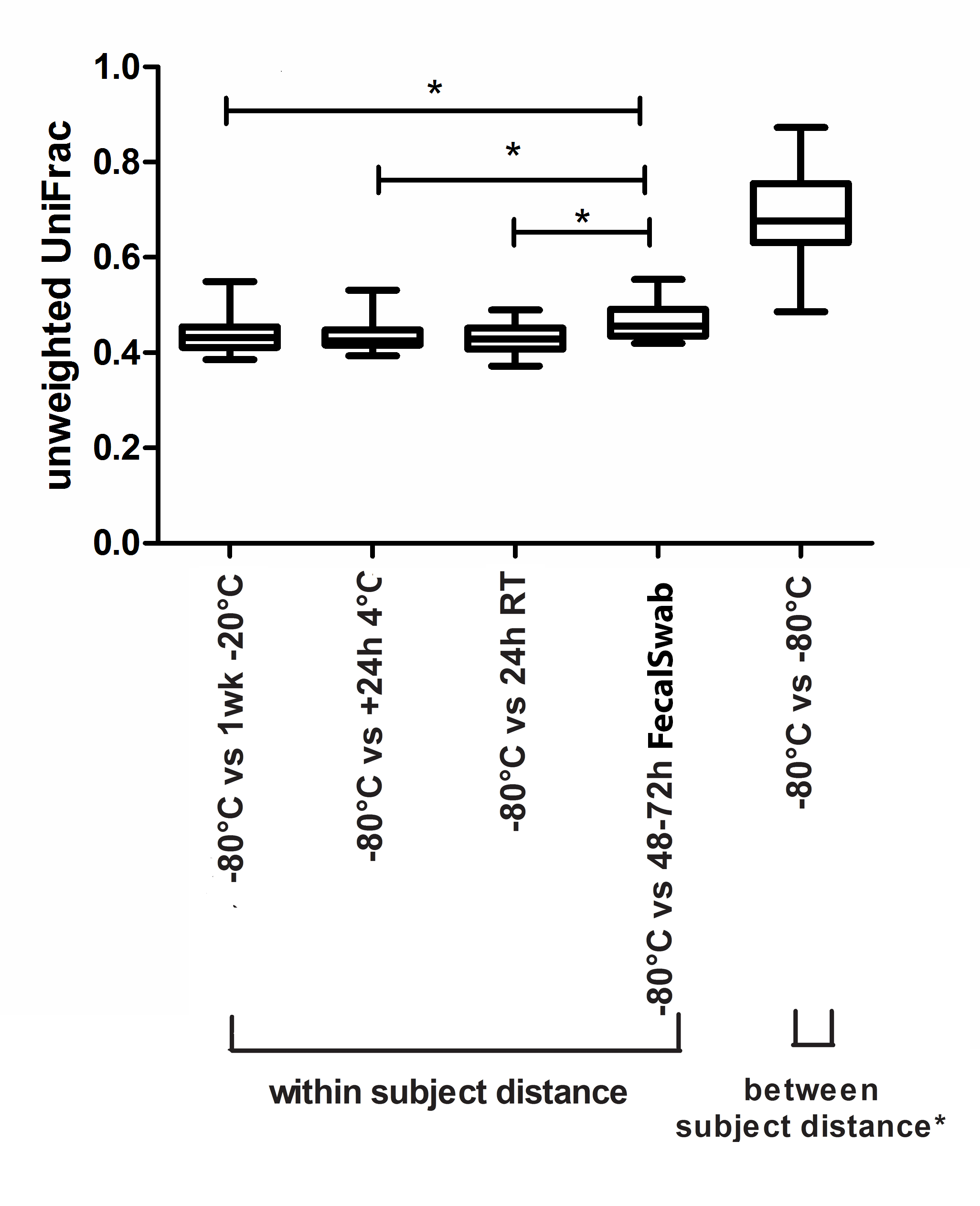

Supplement: S13 Fig — (*p<0.05). (TIF) [file pone.0126685.s013.tif]

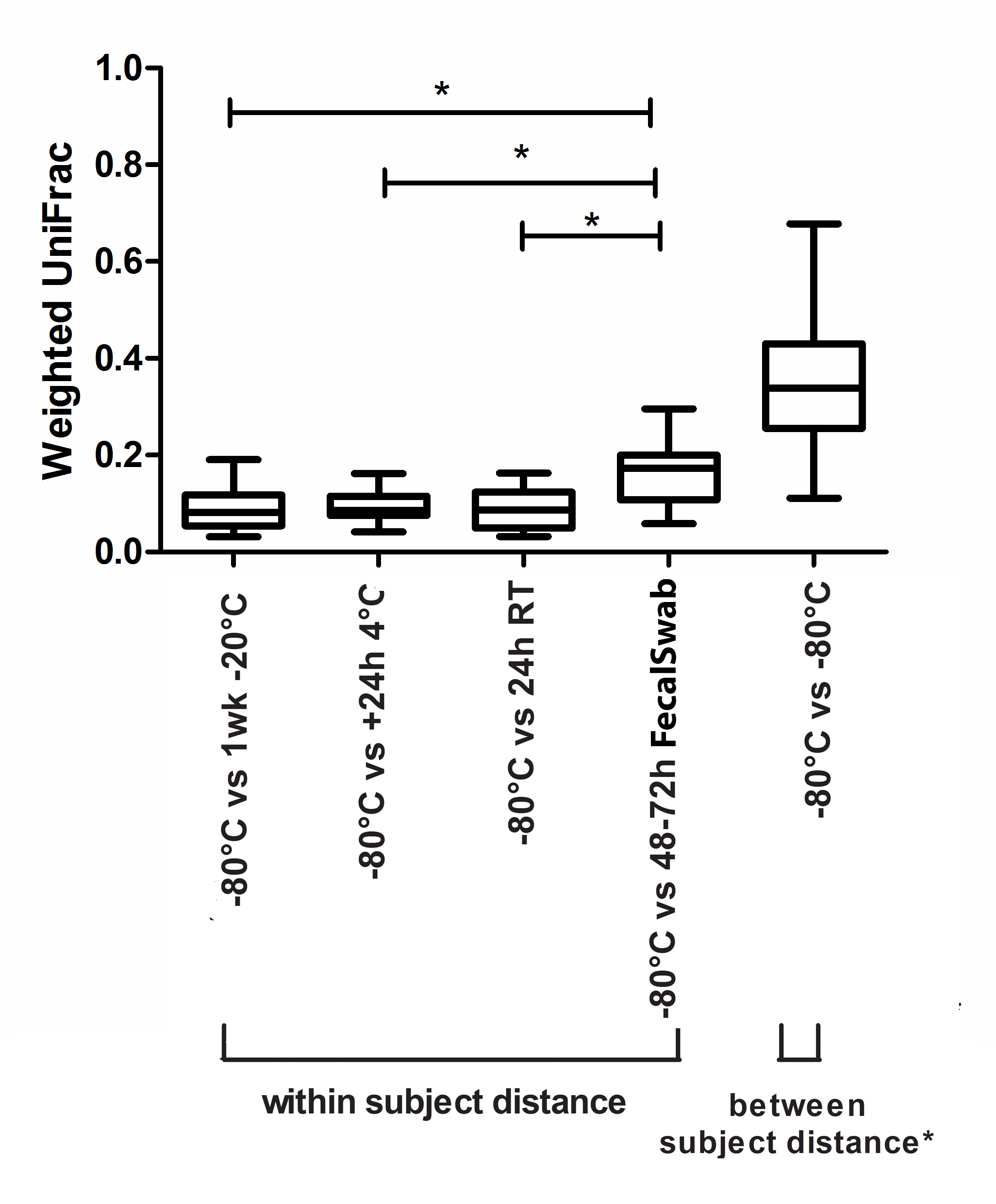

Supplement: S14 Fig — (*p<0.05). (TIF) [file pone.0126685.s014.tif]

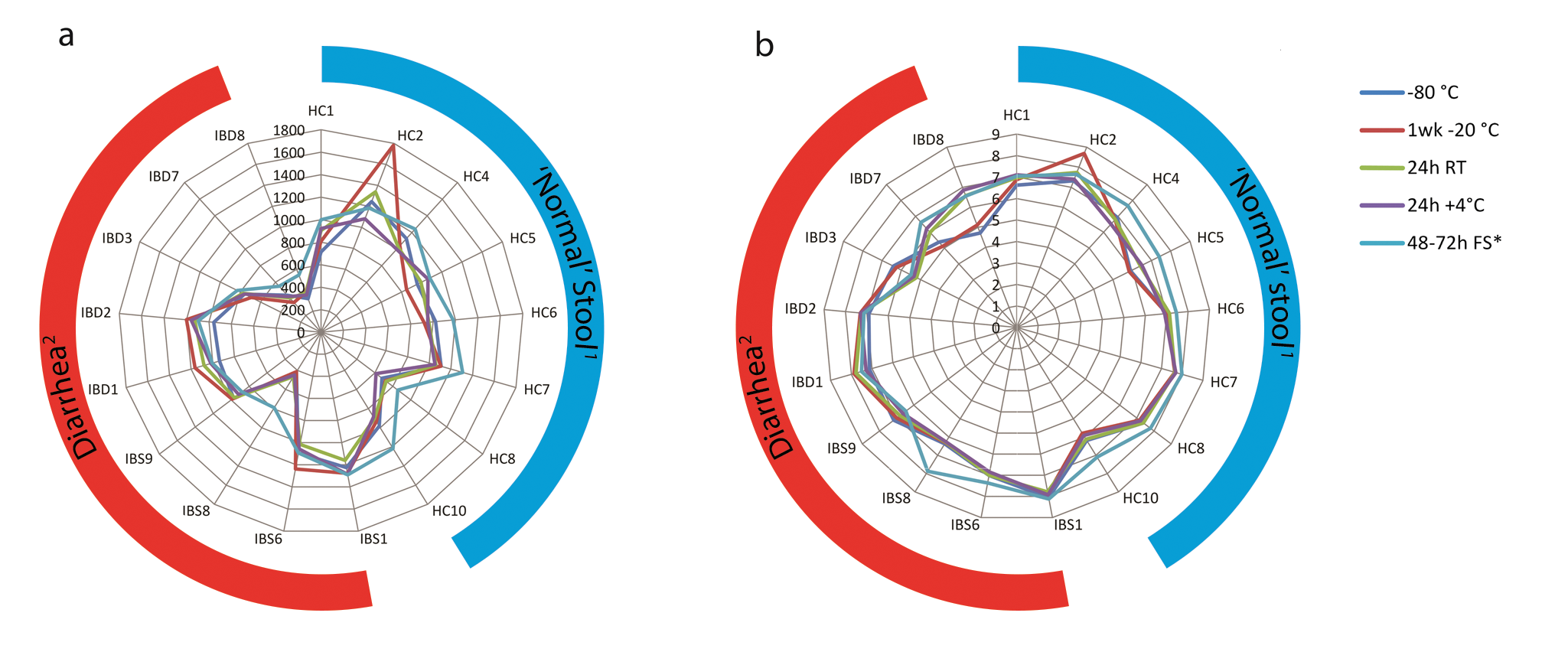

Supplement: S15 Fig — Chao 1 richness estimate (a) and Shannon index (b) is shown at the y axis and test subjects are shown at the x axis. The blue and red bands indicate subjects with normal stool and subjects with diarrhea respectively. Only test subjects (17/28) with a complete set of samples (5 different sampling and storage methods) available for analysis are shown. *p<0.05 comparison diarrhea versus normal stool. 1Bristol stool scale 3–4. 2Bristol stool scale 5–7. (TIF) [file pone.0126685.s015.tif]
