# Supplementary material for: The Effect of Sampling and Storage on the Fecal Microbiota Composition in Healthy and Diseased Subjects
Source: PLoS One. 2015 May 29;10(5):e0126685. doi: 10.1371/journal.pone.0126685 (PMC4449036; doi:10.1371/journal.pone.0126685)
Supplement: S1 Table — Median and range are shown in the table. (*p<0.05 compared to -80°C). (DOCX) [file pone.0126685.s016.docx]

| **Diversity indices** | **Sampling & storage methods** | | | | |
| --- | --- | --- | --- | --- | --- |
|  | **-80 °C** (n=25) | **1w -20 °C** (n=21) | **24h RT** (n=22) | **24h +4°C** (n=22) | **48-72h FecalSwab** (n=13) |
| **Observed species** | 699.0 (206.9-976.1) | 691.8 (176.1-1212.4) | 680.0 (244.6-1001.2) | 662.2 (240.9-929.5) | 760.1^*^ (333.4-906.6) |
| **Chao1** | 1176.0 (349.7-1683.5) | 1202.3 (239.4-2039.6) | 1135.8 (408.4-1816.9) | 1182.6 (394.9-1575.6) | 1271.2^*^ (651.7-1522.5) |
| **Shannon** | 7.0 (4.5-8.5) | 7.0 (4.1-8.8) | 7.1 (5.1-8.5) | 6.9 (4.7-8.2) | 7.2^*^ (5.4-8.2) |
| **PD whole tree** | 32.5 (10.5-42.0) | 32.6 (9.1-52.9) | 32.4 (11.7-43.0) | 32.1 (12.0-40.6) | 34.9^*^ (15.4-42.4) |

**S1 Table. Effect of sampling and storage methods on alpha diversity metrics at a sampling depth of 5000 sequences/sample.** Median and range are shown in the table. (*p<0.05 compared to -80°C)
